# Are Brachycephalic Dogs Really Cute? Evidence from Online Descriptions

**DOI:** 10.1080/08927936.2023.2176590

**Published:** 2023-02-27

**Authors:** Elizabeth S. Paul, Emily Coombe, Paul D. McGreevy, Rowena M. A. Packer, Vikki Neville

**Affiliations:** a Bristol Veterinary School, University of Bristol, Langford, UK; b School of Environmental and Rural Science, Faculty of Science, Agriculture, Business and Law, University of New England, Armidale, Australia; c Department of Clinical Sciences and Services, The Royal Veterinary College, Hatfield, Hertfordshire, UK

**Keywords:** Brachycephalic, cute, dog, human–animal interaction, kindchenschema

## Abstract

Brachycephalic dog breeds have become increasingly popular in recent years, despite showing a high incidence of conformation-related disorders and early mortality. It has been suggested that this popularity might be explained by public perceptions of these short-muzzled dogs as looking particularly infant-like or “cute.” Here, the hypothesis that short-muzzled breeds are especially likely to be described as cute was investigated by analyzing the word contents of advertisements for dogs and puppies being sold online. The ages and breeds of dogs being advertised were considered, in addition to whether the text of each advertisement included the word “cute” or two associated words: “adorable” and “sweet.” Analyses of the entire sample of advertisements (*n* = 43,312) indicated that younger dogs were more likely to be advertised as “cute” and “adorable,” while older ones were more likely to be advertised as “sweet.” Shortmuzzled, brachycephalic breeds (cranio-facial ratio < 0.5) were more likely to be advertised as “cute,” with brachycephalic puppies under 6 months of age being particularly likely to be called “cute” and also “adorable.” However, breed size had a larger and wider effect on word use in advertisements, with smaller dogs being advertised more frequently using all three words: “cute,” “adorable,” and “sweet.” When data for adult dogs only were considered (*n* = 11,400), and continuous muzzle shortening and age data were used, a somewhat different and more complex pattern of results were found. Use of the words “cute” and “adorable” were not associated with degree of muzzle shortening among these adult dogs, but “sweet” was used more often in advertisements for longer-muzzled breeds. We conclude that the present dataset provides partial support for the assertion that short-muzzled dogs are described as more “cute” than longer-muzzled ones, but that small size is a better predictor of the use of “cute” and its synonyms.

Several popular dog breeds, including Pugs, Bulldogs, French bulldogs, and Boston terriers, have flattened faces and extremely short muzzles. Yet this breed-related brachycephalia has become widely recognized as an important constraint on dog health and welfare (Ekenstedt et al., 2020; Farrow et al., 2014; Hendricks, 1992; Liu et al., 2017; Njikam et al., 2009; O’Neill et al., 2015; O’Neill et al., 2017; O’Neill et al., 2019; O’Neill, Packer, et al., 2020; O’Neill, Pegram, et al., 2020; Packer, Hendricks, & Burn, 2015; Packer, Hendricks, Tivers, & Burn, 2015; Packer et al., 2019; Roedler et al., 2013; Seppanen et al., 2019). Numerous professional bodies, charities, and pressure groups have high-lighted breed-related brachycephalia as a serious animal welfare problem (Asher et al., 2009; Bateson, 2016; Brachycephalic Working Group, 2018; British Veterinary Association, 2020a, 2020b; Rooney & Sargan, 2009). Yet, across a similar period, there has been an unprecedented increase in the ownership of several short-muzzled breeds and types (e.g., Teng et al., 2016). Data collected from a random sample of veterinary clinic-attending dogs in 2016 estimated brachycephalic breeds to represent over 18% of the total UK dog population (O’Neill, Packer, et al., 2020). And data from a range of kennel clubs around the world indicate high levels of registration of such dogs, with breeds including the French bulldog and Pug becoming particularly popular (e.g., American Kennel Club, 2021; Australian National Kennel Council, 2021; Centrale Canine, 2021; Japan Kennel Club, 2021; The Kennel Club, 2021). It appears that the association between brachycephalic skull configurations and serious health problems has not deterred the acquisition of short-muzzled dogs. For example, in a recent survey, Packer et al. (2017) found that the factor that most highly influenced owners’ decisions to buy such dogs was “appearance,” with the perceived health of the breed being less influential in decision-making for purchasers of brachycephalic breeds, when compared with those of non-brachycephalic breeds. But what exactly is it about the appearance of these dogs that is so attractive to prospective owners? In the current study, we investigated the hypothesis that breed-related brachycephalia influences people’s descriptions of dogs and puppies as “cute” – as attractive or appealing in an infantile manner (Oxford Languages, 2010).

## The “Cute” Effect

Lorenz (1943, 1971) introduced the term “kindchenschema,” also known as “the cute effect”, to describe the phenomenon whereby the physical characteristics of human infants, such as their small size, disproportionately large heads, prominent foreheads, soft skin, large low-set eyes, and a small nose and chin, serve to automatically elicit affectionate, protective, nurturant, and non-threatening behavior in observers (e.g., Alley, 1981; Almanza-Sepdulveda et al., 2018; Glocker et al., 2009; Hildebrandt & Fitzgerald, 1979). Furthermore, he pointed out that many young nonhuman animals share several of the infantile features characteristic of human infants and that these could also trigger such behavior across species (Lorenz, 1971; see also Caria et al., 2012).

Since the seminal writings of Lorenz, studies of the cute effect have considered the impact of observing infant-like features on people’s behavior and emotions in a wide range of contexts. This research confirms that both photographs and line drawings of human infants can generate affectionate feelings and stimulate attentional and physiological responsiveness in people (Alley, 1983a, 1983b; Esposito et al., 2015; Hildebrandt & Fitzgerald, 1978; Parsons et al., 2011; Power et al., 1982). Images of babies and young children with exaggerated infantile facial features also tend to be preferred by observers and described or rated by Anglophones as more “cute” (Alley, 1981; Fullard & Reiling, 1976; Hildebrandt & Fitzgerald, 1979). Experimental studies in which images are systematically modified according to kindchenschema criteria have also confirmed some of the key facial features originally thought to determine the attractive, nurturance-inducing appearance of the young: a relatively large, prominent forehead and a relatively smaller lower-face region (i.e., small nose, mouth, chin; Alley, 1981; Almanza-Sepdulveda et al., 2018; Glocker et al., 2009). Several such studies provide evidence that relatively large eyes contribute to the cute effect (e.g., Hildebrandt & Fitzgerald, 1979; Sternglanz et al., 1977), although this finding has not been universal (e.g., Almanza-Sepdulveda et al., 2018).

Research into the cute effect using animal images as stimuli has produced broadly similar results. People often show preferences for mammalian young, such as puppies and kittens, rather than older animals (Archer & Monton, 2011; Lehmann et al., 2013), and for animal images modified to have infant-like facial features (e.g., Little, 2012). Archer and Monton (2011) found that dog, cat, and teddy bear faces classified as being higher in kindchenschema facial features were rated more positively by observers. Hecht and Horowitz (2015) demonstrated that modifying facial images of dogs and cats to look more infantile, in particular by increasing relative eye size (and eye separation), significantly influenced people’s preferences. And Borgi et al. (2014) also used manipulated images of dogs and cats, finding that infantile features significantly influenced cuteness ratings and gaze durations from both child and adult observers. Even the features of the young of some non-mammalian species, including reptiles, birds, and invertebrates, can trigger the cute effect (e.g., Kruger, 2015; Miller, 2011). Indeed, infantile images of various animals have also been associated with increased behavioral carefulness in observers, presumed to be a component of nurturant and caregiving behavior (Nittono et al., 2012; Sherman et al., 2009).

## Online Pet Sales and Use of the Word “Cute”

To investigate contemporary observers’ descriptions of the cuteness of different dog breeds, including brachycephalic breeds, we made use of a naturally occurring dataset of online dog and puppy advertisements from a UK pet sales website. Large online datasets, such as this, provide good opportunities to test hypotheses concerning how people describe a range of issues or objects (Golder & Macy, 2014; Nakamura et al., 2020). Here, by identifying which breed was being sold in each advertisement, and identifying whether key words such as “cute” appeared in the sales description, we could establish whether word use varied significantly according to breed and to breed-related factors, such as brachycephalia. The 30 breeds of dog most commonly advertised were considered, and we sought to discover whether shorter-muzzled breeds were more often described in advertisements using “cute” and related endearing terms.

## Breed Brachycephalia and Cuteness

**Figure 1:**
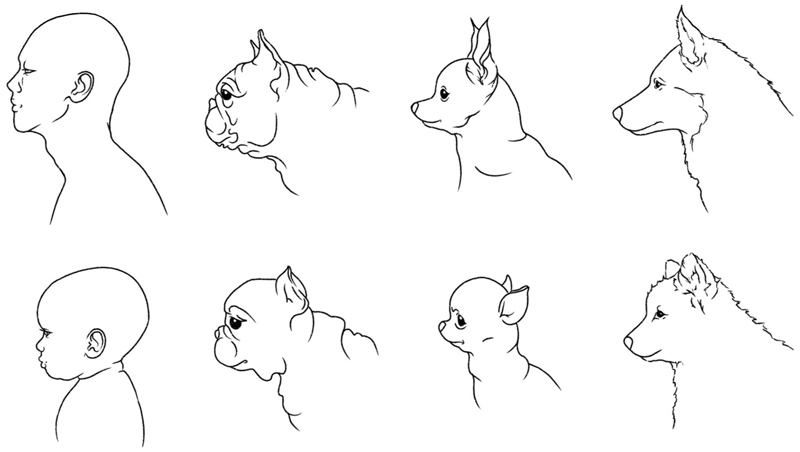
Line drawings illustrating the cranial profiles of adult and infant humans and dogs (left to right: human, French bulldog, Chihuahua, Siberian husky; top to bottom: adult, infant). The two brachycephalic breeds (French bulldog, Chihuahua), although somewhat different in conformation, show a degree of infant-like lower-face reduction in adulthood as well as puppyhood.

In a large, global survey of people’s preferences for images of rabbits of varying face shape, Harvey et al. (2019) found a consistent preference for mildly flat-faced breeds, with the longest-faced rabbits being least preferred. In a similar study of cat-face shapes, however, people’s preferences were less clear and varied according to their animal experience and culture (Farnworth et al., 2018). With regard to dogs, it has frequently been suggested that a key driver of the popularity of short-muzzled breeds is a preference for their perceived cuteness (e.g., McGreevy et al., 2013; Packer, 2021; Packer et al., 2017; Sandøe et al., 2017; Serpell, 2002). Specifically, the suggestion is that the brachycephalic morphology in dogs mimics the features of human (and other mammalian) infants and that such stimuli elicit emotional feelings and behaviors associated with parental love, care, and nurturance, in alignment with the kindchenschema proposal of Lorenz (1943, 1971). It can be seen from Figure 1 that the lower-set nose, mouth, and chin of human infants, and their larger, more prominent forehead, are somewhat mirrored in brachycephalic breeds of dog, even into adulthood. So, the prospect that short-muzzled dogs might be regarded as more “cute” by potential owners is a plausible one. However, the possibility of an association between muzzle shortening and people’s descriptions of dogs and puppies as “cute” has never been investigated empirically.

## Breed Size and Cuteness

Of course, other factors beyond relative muzzle length may also play a part in determining whether people regard certain breeds as especially “cute,” in the sense of being physically appealing in an infantile manner. In particular, the size of a breed may also have an effect. For example, several of the best-known and most popular brachycephalic breeds are small “lap dogs,” which are easy to pick up and cuddle. Indeed, in a recent study, owners of some of these small breeds reported valuing the physical closeness that they could achieve in this way with their pets (Packer et al., 2020). Their small size alone may account for them being regarded as more cute (Alley, 1983a, 1983c; Robson & Moss, 1970). Being small is probably the single most distinctive morphological marker of infancy in humans, and across contemporary dog breeds there is exceptional variation in stature (e.g., Maltese breed standard, 25 cm height at withers; Great Dane 75cm+; The Kennel Club, 2020a, 2020b). So, small size alone may be as good a predictor of the use of words such as “cute” as brachycephalia alone, or the two factors together may interact.

## Methods

### Advertisement Sample

Data from advertisements for the private sale of dogs and puppies on the UK-based website *Preloved* (www.Preloved.co.uk) were extracted across a year-long period (from the beginning of October 2016 to the end of September 2017) using internet bots (a method previously developed by Neville and colleagues for use in a study of rabbits: Neville et al., 2019). Information obtained from each advert included the breed of dog (s) being sold, the dog’s/(dogs’) age (in weeks), the advertisement title and the sale description (usually a short paragraph of five to ten sentences), and the total number of words included in each title and description. In total, 69,917 advertisements concerning the sale of adult dogs and puppies, single dogs and multiples, single-breed dogs, cross-bred dogs, and mongrels were retrieved. From these, those pertaining to the 30 most commonly advertised single breeds (as recognized by The UK Kennel Club) were selected for further analysis; the resultant pool comprised a total of 43,353 advertisements, with more than 300 advertisements per breed. Following removal of dogs whose ages were unavailable or mislabeled, and cases that concerned missing, stolen, or lost dogs, 43,312 advertisements remained. Of these, 11,400 were for adult dogs (defined for the purposes of this study as 6 months of age and over) and 31,912 for puppies (under 6 months of age). Table 1 lists all 30 breeds considered and indicates the number of advertisements sampled for each.

### Adjective Groupings

Words contained in the advertisement titles and descriptions for each breed were run through a frequency counter created in RStudio (R Core Team, 2013). From the resulting list of unique words that occurred in 100 or more of the advertisements, all non-descriptive words were manually removed (e.g., sell, buy, and, he, she, collect, etc.). Misspelt words were pooled (e.g., cutte, kute), as were word extensions (e.g., “cute” also incorporated cutey, cutie, cutest). This left a list of 120 unique adjectives used in the advertisements to describe the dogs’ physical appearance (e.g., beautiful, stunning, fluffy, chunky), their behavior (e.g., affectionate, active, lively), and their more general nature (e.g., wonderful, fun, healthy, super). Two independent observers identified two words from this list, in addition to “cute,” that could be considered to be related to or synonymous with the word “cute” within British vernacular usage: “adorable” and “sweet” (also incorporating “sweetie” and “sweetheart”). Note that in British usage, the meaning of “cute” is strongly focused on looks that are attractive in an appealingly infantile manner (i.e., attractive or pretty in an endearing way); cute is rarely or never used in the sense of clever, cunning, or sexy (Stevenson, 2010). For each advertisement, the appearances of “cute,” “adorable,” or “sweet” in the title and/or in description sections of advertisements were recorded as binary dependent variables (present/absent).

### Relative Muzzle Length and Brachycephalia Classification

The 30 breeds considered here were classified as either brachycephalic or non-brachycephalic on the basis of two published datasets of breed-based measurements of relative muzzle length. The cranio-facial ratio (CFR) is calculated in live dogs by using a soft tape to measure head size. Dividing the muzzle length by the cranial length produces a measure in which lower values represent shorter-muzzled, more brachycephalic morphologies (Sutter et al., 2008). Packer, Hendricks, & Burn (2015) calculated the CFR for 700 dogs across 97 breeds. The cephalic index (CI) is calculated by dividing the anterior-posterior length of the skull by its width at its widest part (x100), producing a measure in which higher values represent shorter-muzzled, more brachycephalic, cranial morphologies (Georgevsky et al., 2014; McGreevy et al., 2004; McGreevy et al., 2013; Roberts et al., 2010). Georgevsky et al. (2014) calculated the CI of 960 dogs across 80 breeds, taking the measurements from standardized images of live adult dogs (six males and six females of each bred) photographed from above (transverse plane of head). Of the 30 breeds considered in the current study, 29 had a mean CFR measure available (Packer, Hendricks, & Burn, 2015) and 23 had a mean CI available (Georgevsky et al., 2014). CFR was therefore used as the primary measure of relative muzzle length (and CI was used as a secondary measure) enabling the classification of brachycephalic dogs with a CFR ≤ 0.5 (see Packer, Hendricks, & Burn, 2015) and non-brachycephalic dogs with a CFR > 0.5.

### Estimated Breed Height and Breed Size Classification

From the UK Kennel Club breed standard descriptions of each breed, withers height (including height ranges for males and females in each breed) were averaged for estimations of the mean height of each of the 30 breeds used in the study, and this was supplemented by American Kennel Club breed standard information for cases where UK estimates were not available (American Kennel Club, 2021; The Kennel Club, 2021). On the basis of these estimations, the 30 breeds considered here were classified as either small or medium/large breeds, using a median split of all breeds’ heights.

### Analyses

To test the hypothesis that breed relative muzzle length (and breed size) would influence word use in advertisements, we first used all cases in the advertisement sample (*n* = 43,312; puppies and adult dogs) to investigate the effects of a range of potential predictor variables on whether “cute,” “adorable,” and “sweet” appeared in the texts of advertisements. Predictor variables considered in the logistic regression were: breed brachycephalia (brachycephalic; non-brachycephalic); breed size (small; medium/large); and dog age (weeks). Two models were run for each adjective: one with and one without interaction terms (age × breed size; age × breed brachycephalia; breed size × breed brachycephalia). It was noted in preliminary analyses that the total number of words used within each advert was significantly higher when “cute,” “adorable,” and “sweet” were present (i.e., there was a positive association between cute-word usage and total word use – Cute: *t*_(43,310)_ = 11.522, *p* < 0.001; Adorable: *t*_(43,310)_ = 16.699, *p* < 0.001; Sweet: *t*_(43,310)_ = 21.356, *p* < 0.001). Because of this, the continuous variable total word count (total number of words used in each advertisement) was also added to the models.

These analyses used conservative, binary classifications of breed size (small; medium/large) and breed relative muzzle length (brachycephalic; non-brachycephalic) because it was not possible to ensure that the full (continuous) measures from which they were derived (breed CFR and breed height) would be reliable across all ages of dogs (see Methods section above). For example, puppies of brachycephalic breeds, while still considerably shorter-muzzled than those of non-brachycephalic breeds, may not show as great a difference in CFR as they do as adults (and measures of mean breed CFR, CI, or mean breed height at withers are not currently available for different ages of puppies; Andreis et al., 2018). We accept that such analyses lose some of the richness of the relative muzzle length and estimated breed height data sets. Therefore, we conducted additional logistic regression analyses for comparison purposes, making use of full (continuous) measures of mean breed CFR (relative muzzle length in the form of mean CFR; Packer, Hendricks, & Burn, 2015) and mean breed height (mean height at withers, cm; American Kennel Club, 2021; The Kennel Club, 2021) as potential predictor variables. These analyses considered only data on adult dogs (age ≥ 6 months), for whom these continuous variables are available. The two predictor variables of breed CFR and breed height were not significantly correlated in the current sample (Pearson’s *r* = 0.309, *n* = 29, *ns*). Age was not included in these models (all cases were of adult dogs, with a strong skew to younger ages), but as above, total word count (total number of words used in each advertisement) was included.

## Results

### Breed Sample

The relative muzzle length (breed CFR; breed CI), size (breed height), brachycephalia classification (brachycephalic; non-brachycephalic), and breed size classification (small; medium/large) of the 30 breeds studied are all shown in Table 2. No CFR data were available for one breed, the Siberian husky, but this was classified as non-brachycephalic on the basis of a CI of 60.92 (Georgevsky et al., 2014).

### “*Cute*”, “*Adorable*,” *and* “*Sweet*”

**Figure 2:**
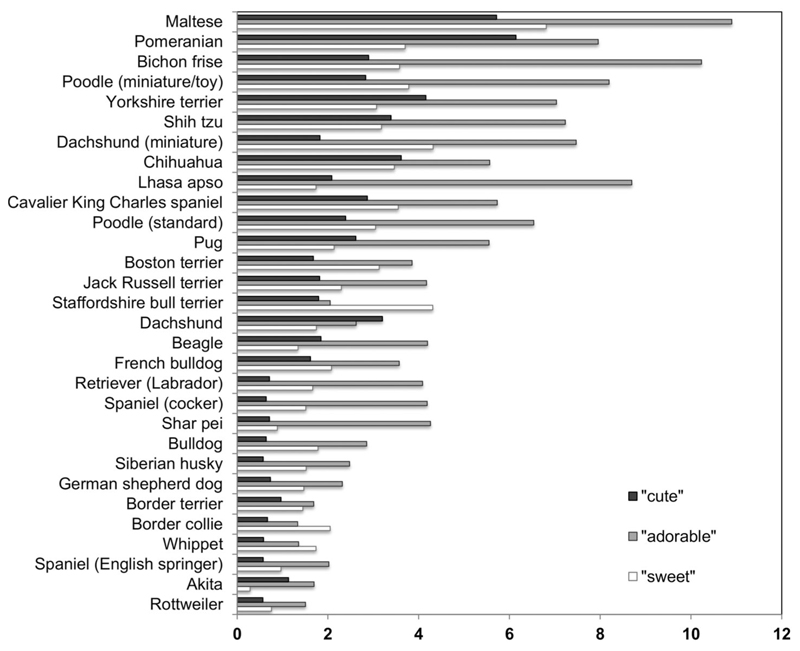
The percentage of all advertisements including the words “cute,” “adorable,” and “sweet.”

Figure 2 shows the percentage of advertisements for each breed that included “cute,” “adorable,” and “sweet.” The word “cute” occurred at a rate of 2.10% across all advertisements (also incorporating “kute,” “cutie,” “cutest”); “sweet” occurred at a similar rate (2.51% of advertisements; also incorporating “sweetie” and “sweetheart”), while “adorable” was most common (4.53% of advertisements).

**Figure 3:**
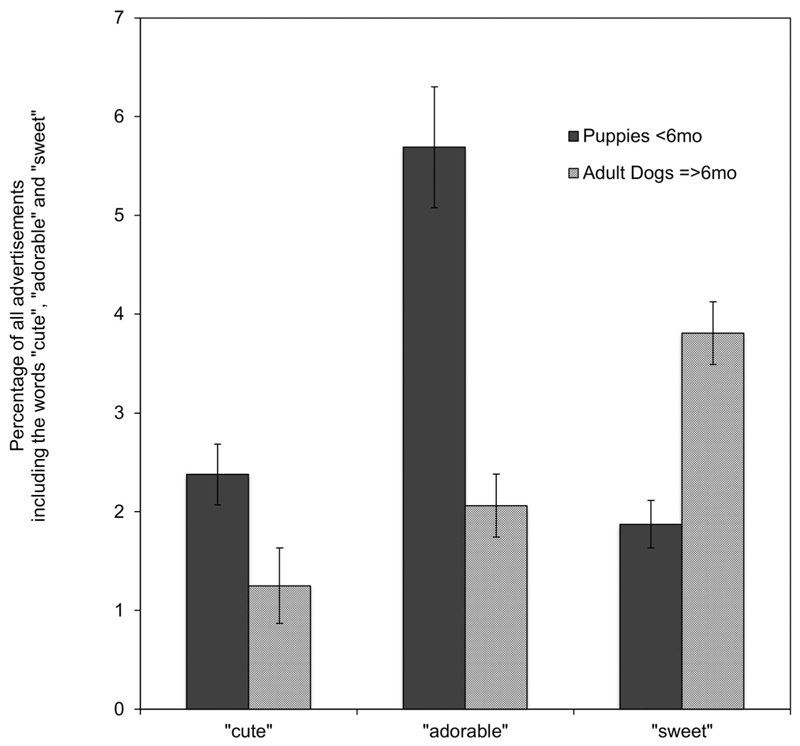
The percentage of advertisements for all breeds of puppies (<6 months) and adult dogs (≥6 months) including the words “cute,” “adorable,” and “sweet.”

Preliminary analyses confirmed that use of the three words (cute, adorable, and sweet) was linked, suggesting semantic similarity. Across the entire sample, “adorable” occurred 3.56 times more often, and “sweet” occurred 3.38 times more often, in advertisements that also contained “cute”, than in those that did not contain “cute” $({\text{X}}_{\left(2\right)}^{2}=248.186,\;p<0.001;{\text{X}}_{\left(2\right)}^{2}=120.332,\;p<0.001)$. However, additional exploratory analyses also suggested some differences in the ways in which these words were used. Most notably, comparison of advertisements for puppies (< 6 months) and adult dogs (≥ 6 months) revealed that, while “cute” and “adorable” were more commonly used in advertisements for puppies, “sweet” was more often used in advertisements for adult dogs. Specifically, 2.5% of advertisements for puppies and 1.1% of advertisements for adults included “cute” $({\text{X}}_{\left(2\right)}^{2}=77.134,p<0.001)$ 5.6% of advertisements for puppies and 1.9% of advertisements for adults included “adorable” $\left(x_{\left(2\right)}^{2}=268.170,\;p<0.001\right);$ and 2.0% of advertisements for puppies and 3.8% of advertisements for adults included “sweet” $\left(x_{\left(2\right)}^{2}=111.937,\;p<0.001\right)$ (Figure 3). Because of this variability in usage, the three words were not combined into a single category (i.e., presence cute and/or adorable and/or sweet) and were instead considered separately in all the analyses presented below.

### Whole Sample: Effects of Dog Age, Breed Size, Breed Brachycephalia, and Total Word Count on Use of “Cute,” “Adorable,” and “Sweet” in Advertisements

Using logistic regression analyses, dog age (weeks), breed size classification (small; medium/large, based on median split of breed height data), breed brachycephalia classification (brachycephalic; non-brachycephalic), and total word count (per advertisement) were investigated as potential predictors of the presence of “cute,” “adorable,” and “sweet.” The results of these analyses are shown in Table 3. As anticipated, total word count had consistently significant but small effects, with all three words being more likely to appear in longer advertisements. Dog age also had a significant main effect on the use of “cute” and “adorable,” with more advertisements for younger dogs including these words; “sweet,” on the other hand, was more often used in advertisements for older dogs (Figure 3).

**Figure 4:**
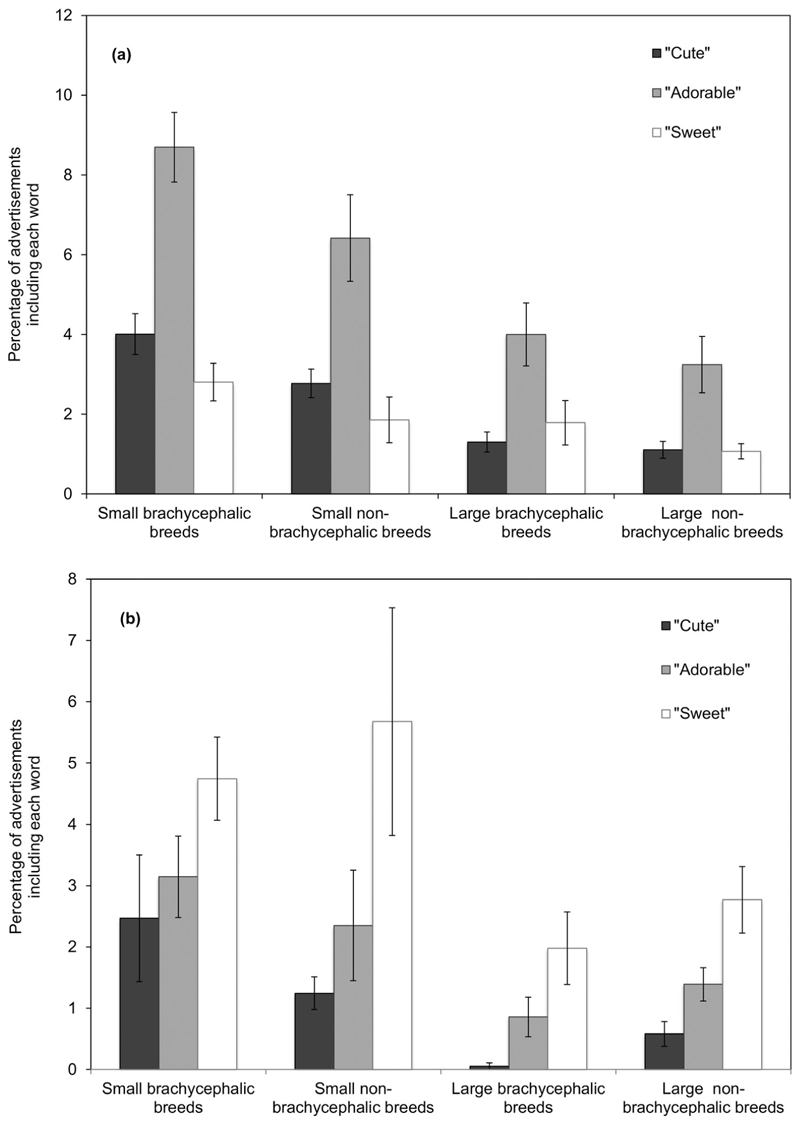
(a) The percentage of advertisements for puppies (< 6 months) classified as small vs. medium/large and brachycephalic/non-brachycephalic, including the words “cute,” “adorable,” and “sweet”; (b) The percentage of advertisements for adult dogs (≥ 6 months) classified as small vs. medium/large and brachycephalic/non-brachycephalic including the words “cute,” “adorable,” and “sweet.”

Brachycephalic dogs were more likely than non-brachycephalic dogs to be advertised using the word “cute,” but not “adorable” or “sweet.” In addition, a significant interaction between brachycephalia classification and age was revealed, with younger brachycephalic dogs being more likely to be described as “cute” or “adorable.” However, breed size had the largest and most consistent effects on word use in advertisements, with smaller dogs being significantly more likely to be advertised using the words “cute,” “adorable,” and “sweet.” Younger dogs and puppies of small breeds were especially likely to be advertised using the word “adorable.” Figure 4(a,b) illustrate these findings.

### Adult Sample Only: Effects of Estimated Breed Height, Mean Breed Cranio-Facial Ratio (CFR), and Total Word Count on Use of “Cute,” “Adorable,” and “Sweet” in Advertisements

A second, complementary set of logistic regressions were used to assess the continuous variables of breed height, breed CFR (relative muzzle length measured using CFR; Packer, Hendricks, & Burn, 2015), and total word count as potential predictors of whether “cute,” “adorable,” or “sweet” were included in advertisements for adult dogs only. The results are summarized in Table 4. As before, total word count used had consistently significant but small effects, with all three words being more likely to appear in longer advertisements. Advertisements for smaller breeds also used “cute,” “adorable,” and “sweet” significantly more often than did those for larger breeds. However, relative muzzle length (CFR) was not significantly associated with use of the words “cute” or “adorable,” but showed a small association with use of “sweet,” with advertisements for shorter-muzzled dogs using this word less, rather than more, often (main effect only).

## Discussion

It has been widely suggested that the popularity of brachycephalic dog breeds might be explained by their cute, infant-like, kindchenschema looks. Such suggestions have come from researchers investigating the detrimental effects of extreme muzzle shortening on canine health and from professional commentators seeking to highlight brachycephalia as a major welfare issue (e.g., British Veterinary Association, 2020a, 2020b; McGreevy et al., 2013; O’Neill et al., 2022; Packer et al., 2017; Sandøe et al., 2017; Serpell, 2002). Evidence of a direct link between muzzle shortening in dogs and perceptions of them by people as “cute,” however, remains elusive. In the current study, we made use of an extensive natural dataset, in the form of online dog-sales advertisements, to investigate whether short muzzles are associated with increased usage of the adjective “cute” and of two closely associated words: “adorable” and “sweet.”

### Dog Age

In the UK, vernacular use of the word “cute” and its synonyms refers to an attractive and endearing, often infant-like, appearance. It was therefore not surprising to find in our analyses (of the entire dataset) that both “cute” and “adorable” occurred significantly more frequently in advertisements for puppies and young dogs; these words were being used to describe infantilism in some form. However, this effect of age was relatively small; these words were sometimes used to describe older dogs, indicating that even these animals may have been regarded as infant-like to some degree. And a presumed synonym of “cute,” “sweet,” was actually more often associated with older dogs. This raises the possibility that this word, despite being definitionally similar to cute, may also have other meanings in this context, such as “pretty” or “pleasant-natured” (Stevenson, 2010). For example, people may use the word “sweet” more commonly in the context of describing the behavior or perceived personality of their dogs. Further research, making more detailed use of the full descriptive phrases used, may help to elucidate this in the future (e.g., using thematic text analyses; Packer et al., 2020).

### Breed Brachycephalia and Relative Muzzle Length

Our primary hypothesis that short-muzzled puppies and dogs would be described more frequently as “cute,” “adorable,” and “sweet” was partially supported. Analyses of the entire dataset of dogs and puppies, which considered a binary classification of brachycephalia as a predictor of word usage (CFR ≤ 0.5 and CFR > 0.5; Packer, Hendricks, & Burn, 2015), showed that the word “cute” did indeed occur more frequently in online advertisements for brachycephalic breeds. In addition, younger dogs of these breeds were advertised more often as “cute” and “adorable” than older dogs were. However, our subsequent analyses, focusing on adult dogs only, found no significant effects of breed CFR on use of either “cute” or “adorable,” while “sweet” appeared more commonly in advertisements for longer-muzzled breeds than shorter-muzzled breeds. These differences between the two sets of analyses can be explained in two ways. First, the division of both muzzle length and breed height into binary variables (brachycephalic/non-brachycephalic breed; small/large breed) must have inevitably had a simplifying effect, perhaps revealing an association between muzzle length and cuteness that was more complex (and less clear-cut) when the full, continuous datasets were used (CFR; mean estimated breed height). Second, there was also a discontinuity across age (i.e., above and below 6 months) in the relationship between breed CFR and descriptions of cuteness, with brachycephalic and non-brachycephalic puppies showing most differences (see Figure 4(a,b)). These latter findings indicate that, particularly among younger dogs, shorter-muzzled breeds do indeed tend to be described as more appealing in an infantile manner. Among older brachycephalic dogs who also show somewhat kindchenschema features (see Figure 1), such a tendency is either weak or absent.

Taken together, our results confirm some degree of association between breed-related muzzle length morphologies and attributions of cuteness. But they do not support the hypothesis that short muzzles in adult dogs are universally attractive or appealing in infantile manner (“cute,” “adorable”). In fact, the finding that advertisements for adult dogs using the word “sweet” were significantly less common for shorter-muzzled breeds may point to some kind of reduction in appeal for brachycephalic dogs in adulthood. There are a number of possible reasons for these mixed findings. For example, it is worth considering the possibility that the associations (and absence of associations) reported here may not be accurate representations of the perceptions and sentiments of actual purchasers and potential owners. It is in the interests of all vendors of puppies and dogs to describe them in an appealing and attractive way; their use of adjectives such as “cute” and “adorable” may be as much related to their motivation to sell as to any commitment to sincerity in the description of their animals. That is, they may not, themselves, perceive these animals as “cute.” Perhaps owners with no ulterior motives (e.g., not selling puppies) would be more likely to describe their brachycephalic or shorter-muzzled pets as “cute” and/or less likely to use these terms for other, longer-muzzled breeds. Certainly, it will be important to find other sources of evidence before any final conclusions can be drawn about owners’ (and prospective owners’) actual perceptions of brachycephalic dogs. For example, there may be aspects of the facial appearance of dogs in general, or of brachycephalic dogs in particular, that moderate the kindchenschema effects commonly seen in human infants (e.g., skin folds and wrinkling).

### Breed Size and Height

The current findings consistently support our supplementary hypothesis: that smaller dogs would be described as “cute,” “adorable,” and “sweet” more frequently than large ones. This finding aligns with previous evidence that small size is a strong marker of perceived infancy and predictor of cuteness attributions (e.g., Alley, 1983a, 1983b; Robson & Moss, 1970). Here, small breed size (categorically defined using a median split of the 30 most popular dog breeds for whom advertisement data were used; see Table 1) was significantly associated with more frequent use of “cute,” “adorable,” and “sweet” across all ages, and younger puppies and dogs that were also small were particularly likely to be advertised using the word “adorable.” Within the adult-only sample, estimated breed height remained a significant inverse predictor of the use of “cute,” “adorable,” and “sweet.” We conclude that the size of each breed in the study had a consistent effect on how individual dogs were described and that this effect was not moderated by degree of muzzle shortening.

This inverse relationship between breed size and use of the word “cute” in advertisements may be driven both directly, by smaller dogs’ more infantile looks, and indirectly, through the type of relationship that owners both choose and develop with smaller dogs (which itself is likely to be influenced by both owner behaviors and dog behaviors, as well as physical perceptions; e.g., see McGreevy et al., 2013; Packer et al., 2019; Packer et al., 2020; Stone et al., 2016). And to the extent that cuteness perceptions may encourage acquisition of smaller breeds, this could well have welfare implications of its own (e.g., see Freyer, 2017). The popularity of advertisements for “teacup” varieties – miniaturized versions of breeds that are already small – was noted when the current dataset was being superficially explored. Certainly, the growing interest in miniaturizing already small breeds, such as the Chihuahua and Cavalier King Charles spaniel, is a welfare concern in its own right that will warrant future monitoring and research (Blue Cross, 2020; O’Neill, Pegram, et al., 2020).

## Conclusion

Small size was a strong and consistent predictor of the word “cute” appearing in descriptions of puppies and dogs advertised online, but brachycephalia (categorically defined) and relative muzzle shortening (continuously measured) had weaker and more complex effects, mostly confined to advertisements for younger animals. This finding is potentially good news for those concerned about the current, exceptional popularity of certain short-muzzled dog breeds and their associated welfare problems (e.g., Farrow et al., 2014; O’Neill et al., 2022). People who own brachycephalic dogs report being attracted to them because of their appearance (Packer et al., 2017), but many have also been found to have an unrealistic appreciation of the severity of their health risks (Packer et al., 2019). If this cannot be easily explained by some fundamental human attraction to short-muzzled dogs (as a result of their cute, infant-like appearance), then perhaps there is a better chance that these breeds’ popularity will fall as quickly as they have risen. On the other hand, the strong and consistent links found here between small breed size and attributions of cuteness point to other potential welfare concerns, with the likely persistence of the popularity of small, and sometimes extremely small, breeds. Future psychological studies of people’s motivations to keep pathologically small and short-muzzled breeds, and of how these motivations might be reduced or moderated, are likely to prove invaluable to the future welfare of companion dogs.

## Figures and Tables

**Table 1:** Total number of online advertisements for each of the 30 most commonly advertised breeds.

Breed	Total number of advertisements (all ages)
Chihuahua	6,551
French bulldog	4,699
Pug	3,365
Retriever (Labrador)	2,518
Spaniel (cocker)	2,504
Shih tzu	2,418
Staffordshire bull terrier	2,388
Border collie	1,948
Jack Russell terrier	1,869
German shepherd dog	1,767
Bulldog	1,401
Pomeranian	1,106
Siberian husky	1,049
English springer spaniel	1,037
Yorkshire terrier	1,009
Cavalier King Charles spaniel	732
Poodle (miniature/toy)	634
Dachshund (miniature)	602
Beagle	595
Bichon frise	586
Lhasa apso	575
Shar pei	563
Rottweiler	529
Whippet	516
Poodle (standard)	459
Boston terrier	415
Border terrier	414
Maltese	367
Akita	353
Dachshund (standard)	343

**Table 2:** The mean breed cranio-facial ratio (CFR; *n* = 29; Packer, Hendricks, Tivers, & Burn, 2015), mean breed cephalic index (CI; *n* = 23; Georgevsky et al., 2014) and breed height* (*n* = 30; American Kennel Club, 2021; The Kennel Club, 2021) of the 30 dog breeds included in the study. Based on these, classifications of each breed in terms of breed brachycephalia (brachycephalic CFR ≤ 0.5; non-brachycephalic CFR > 0.5) and breed size (median split of estimated breed height at withers) are also shown.

Breed	Mean breed CFR	Mean breed CI	Breed height	Brachycephaly	Breed size
Pug	0.08	95.68	30.48	B	S
Boston terrier	0.15	83.27	40.64	B	ML
French bulldog	0.18	105.34	30.48	B	S
Shih tzu	0.21	86.34	27.00	B	S
Bulldog	0.22	88.72	36.83	B	ML
Maltese	0.35	63.85	25.00	B	S
Chihuahua	0.35	67.44	16.51	B	S
Lhasa apso	0.38		25.00	B	S
Border terrier	0.39		34.29	B	ML
Cavalier King Charles spaniel	0.40	74.52	31.75	B	S
Pomeranian	0.41	77.57	16.51	B	S
Yorkshire terrier	0.46		19.05	B	S
Bichon frise	0.47	70.78	25.50	B	S
Shar pei	0.47		48.50	B	ML
Staffordshire bull terrier	0.51	75.47	38.50	NB	ML
Rottweiler	0.52	69.42	63.50	NB	ML
Akita	0.53	59.60	68.50	NB	ML
Spaniel (cocker)	0.55	44.28	39.25	NB	ML
Spaniel (English springer)	0.55	51.59	51.00	NB	ML
Jack Russell terrier	0.56	64.52	27.50	NB	S
Beagle	0.56	66.49	33.00	NB	S
Poodle (miniature/toy)	0.57		33.00	NB	S
Retriever (Labrador)	0.58	54.73	56.00	NB	ML
Dachshund (miniature)	0.62	47.65	13.97	NB	S
Border collie	0.67	57.85	53.00	NB	ML
Poodle (standard)	0.69		38.00	NB	ML
German shepherd dog	0.71	55.54	60.50	NB	ML
Dachshund	0.72		21.59	NB	S
Whippet	0.87	47.85	47.25	NB	ML
Siberian husky		60.92	55.00	NB	ML

* Breed height = estimated mean at withers (cm); B = brachycephalic; NB = non-brachycephalic; S = small; ML = medium/large.

**Table 3:** Results of multiple logistic regressions to investigate the effects of dog age, breed size (small; medium/large), breed brachycephalia classification (brachycephalic, cranio-facial ratio CFR ≤ 0.5; non-brachycephalic, CFR > 0.5), and total word count (used in advertisement) on the proportion of advertisements for each breed containing words “cute,” “adorable,” and “sweet” (*n* = 43,312).

“Cute”				
Model 1		95% Confidence interval for odds ratio		
	*B* (*SE*)	Lower	Odds ratio	Upper
Constant	−3.691 (0.054) *p* < 0.001			
Dog age	−0.002 (0.001) *p* < 0.01	0.997	0.998	0.999
Breed size (S; M/L)	−1.061 (0.111) *p* < 0.001	0.278	0.346	0.430
Brachycephalic (Y/N)	−0.243 (0.099) *p* < 0.05	0.784	0.646	0.951
Total word count	0.003 (0.000) *p* < 0.001	1.002	1.003	1.003
*R*^2^ = 0.008 (Cox & Snell); 0.043 (Nagelkerke); χ^2^ = 345.153, *p* < 0.001				
Model 2 (including interactions)			95% Confidence interval for odds ratio	
	*B* (*SE*)	Lower	Odds ratio	Upper
Constant	−3.625 (0.056) *p* < 0.001			
Dog age	−0.004 (0.001) *p* < 0.001	0.994	0.996	0.998
Breed size (*S*; M/L)	−1.327 (0.212) *p* < 0.001	0.175	0.265	0.402
Brachycephalic (Y/N)	−0.468 (0.129) *p* < 0.001	0.486	0.626	0.807
Total word count	0.002 (0.000) *p* < 0.001	1.002	1.002	1.003
Dog age × breed size	0.000 (0.001) *ns*	0.997	1.000	1.003
Breed size × brachycephalic	0.405 (0.254) *ns*	0.912	1.500	2.468
Brachycephalic × age	0.004 (0.001) *p* < 0.01	1.001	1.004	1.007
*R*^2^ = 0.008 (Cox & Snell); 0.045 (Nagelkerke); χ^2^ = 359.489, *p* < 0.001				
“Adorable"				
Model 1			95% Confidence interval for odds ratio	
	*B* (*SE*)	Lower	Odds ratio	Upper
Constant	−3.012 (0.039) *p* < 0.001			
Dog age	−0.004 (0.001) *p* < 0.001	0.995	0.996	0.997
Breed size (*S*; M/L)	−0.688 (0.069) *p* < 0.001	0.439	0.503	0.576
Brachycephalic (Y/N)	−0.039 (0.065) *ns*	0.847	0.962	1.093
Total word count	0.003 (0.000) *p* < 0.001	1.003	1.003	1.003
*R*^2^ = 0.012 (Cox & Snell); 0.038 (Nagelkerke); χ2 = 512.419, *p* < 0.001				
Model 2 (including interactions)			95% Confidence interval for odds ratio	
	*B* (*BE*)	Lower	Odds ratio	Upper
Constant	−2.919 (0.042) *p* < 0.001			
Dog age	−0.009 (0.001) *p* < 0.001	0.989	0.991	0.993
Breed size (*S*; M/L)	−0.737 (0.117) *p* < 0.001	0.381	0.479	0.602
Brachycephalic (Y/N)	−0.120 (0.084) *ns*	0.752	0.887	1.046
Total word count	0.003 (0.000) *p* < 0.001	1.002	1.003	1.003
Dog age × breed size	0.003 (0.001) *p* < 0.01	1.001	1.003	1.006
Breed size x brachycephalic	−0.069 (0.0144) *ns*	0.704	0.933	1.237
Brachycephalic × dog age	0.005 (0.001) *p* < 0.01	1.002	1.005	1.007
*R*^2^ = 0.013 (Cox & Snell); 0.042 (Nagelkerke); χ^2^ = 559.725, *p* < 0.001				
“Sweet"				
Model 1			95% Confidence interval for odds ratio	
	*B* (*BE*)	Lower	Odds ratio	Upper
Constant	−4.089 (0.051) *p* < 0.001			
Dog age	0.004 (0.000) *p* < 0.001	1.003	1.004	1.004
Breed size (*S*; M/L)	−0.475 (0.089) *p* < 0.001	0.523	0.622	0.740
Brachycephalic (Y/N)	−0.081 (0.086) *ns*	0.779	0.922	1.092
Total word count	0.004 (0.000) *p* < 0.001	1.003	1.004	1.004
*R*^2^ = 0.010 (Cox & Snell); 0.047 (Nagelkerke); χ^2^ = 429.203, *p* < 0.001				
Model 2 (including interactions)			95% Confidence interval for odds ratio	
	*B* (*BE*)	Lower	Odds ratio	Upper
Constant	−4.036 (0.055) *p* < 0.001			
Dog age	0.003 (0.000) *p* < 0.001	1.002	1.003	1.004
Breed size (*S*; M/L)	−0.666 (0.155) *p* < 0.001	0.379	0.514	0.697
Brachycephalic (Y/N)	−0.185 (0.121) *ns*	0.655	0.831	1.054
Total word count	0.004 (0.000) *p* < 0.001	1.003	1.004	1.004
Dog age × breed size	0.001 (0.001) *ns*	1.000	1.001	1.002
Breed size × brachycephalic	0.203 (0.192) *ns*	0.841	1.225	1.784
Brachycephalic × dog age	0.001 (0.001) *ns*	0.999	1.001	1002
*R*^2^ = 0.010 (Cox & Snell); 0.048 (Nagelkerke); χ^2^ = 436.964, *p* < 0.001				

**Table 4:** Results of multiple logistic regressions to investigate the effects of breed height (cm; American Kennel Club, 2021; The Kennel Club, 2021), breed cranio-facial ratio (CFR) (Packer, Hendricks, Tivers, & Burn, 2015), and total words (used in advertisement) on the proportion of advertisements for each breed containing words “cute,” “adorable,” and “sweet” (*n* = 11,400).

“Cute”				
Model 1	*B* (*SE*)	95% Confidence interval for odds ratio		
		Lower	Odds ratio	Upper
Constant	−3.303 (0.268) *p* < 0.001			
Breed height	−0.042 (0.008) *p* < 0.001	0.944	0.959	0.973
Breed CFR	−0.059 (0.557) ns	0.317	0.943	2.808
Total word count	0.002 (0.001) p< 0.001	1.001	1.002	1.003
*R*^2^ = 0.005 (Cox & Snell); 0.041(Nagelkerke); χ^2^ = 57.809, *p* < 0.001				
Model 2 (including interactions)		95% Confidence interval for odds ratio		
	*B* (*SE*)	Lower	Odds ratio	Upper
Constant	−4.119 (0.950) *p* < 0.001			
Breed height	−0.016 (0.031) ns	0.927	0.985	1.046
Breed CFR	1.703 (2.035) ns	0.102	5.488	296.076
Total word count	0.002 (0.001) p< 0.001	1.001	1.002	1.003
Breed height × breed CFR	−0.054 (0.061) ns	0.841	0.947	1.067
*R*^2^ = 0.005 (Cox & Snell); 0.041(Nagelkerke); χ^2^ = 58.619, *p* < 0.001				
“Adorable”				
Model 1		95% Confidence interval for odds ratio		
	*B* (*SE*)	Lower	Odds ratio	Upper
Constant	−3.566 (0.202) *p* < 0.001			
Breed height	−0.030 (0.006) *p* < 0.001	0.959	0.970	0.981
Breed CFR	−0.634 (0.439) ns	0.797	1.885	4.456
Total word count	0.004 (0.000) *p* < 0.001	1.003	1.004	1.005
*R*^2^ = 0.007 (Cox & Snell); 0.039 (Nagelkerke); χ^2^ = 82.329, *p* < 0.001				
Model 2 (including interactions)		95% Confidence interval for odds ratio		
	*B* (*SE*)	Lower	Odds ratio	Upper
Constant	−3.251 (0.696) *p* < 0.001			
Breed height	−0.041 (0.022) *p* = 0.066	0.920	0.960	1.003
Breed CFR	−0.020 (1.453) ns	0.057	0.980	16.926
Total word count	0.004 (0.000) *p* < 0.001	1.003	1.004	1.005
Breed height x breed CFR	0.020 (0.041) ns	0.941	1.020	1.105
*R*^2^ = 0.007 (Cox & Snell); 0.039 (Nagelkerke); χ^2^ = 82.552, *p* < 0.001				
“Sweet”				
Model 1		95% Confidence interval for odds ratio		
	*B* (*SE*)	Lower	Odds ratio	Upper
Constant	−3.199 (0.150) *p* < 0.001			
Breed height	−0.029 (0.004) *p* < 0.001	0.963	0.971	0.979
Breed CFR	1.145 (0.328) *p* < 0.001	1.653	3.143	5.975
Total word count	0.004 (0.000) *p* < 0.001	1.003	1.004	1.005
*R*^2^ = 0.013 (Cox & Snell); 0.046 (Nagelkerke); χ^2^ = 150.295, *p* < 0.001				
Model 2 (including interactions)		95% Confidence interval for odds ratio		
	*B* (*SE*)	Lower	Odds ratio	Upper
Constant	−3.609 (0.510) *p* < 0.001			
Breed height	−0.016 (0.016) ns	0.953	0.984	1.015
Breed CFR	1.983 (1.045) *p* = 0.058	0.938	7.265	56.295
Total word count	0.004 (0.000) *p* < 0.001	1.003	1.004	1.005
Breed height x breed CFR	−0.025 (0.030) ns	0.920	0.975	1.034
*R*^2^ = 0.013 (Cox & Snell); 0.046 (Nagelkerke); χ^2^ = 151.009, *p* < 0.001				

*ns* = not significant.
